# The Relationships of Markers of Cholesterol Homeostasis with Carotid Intima-Media Thickness

**DOI:** 10.1371/journal.pone.0013467

**Published:** 2010-10-18

**Authors:** Oliver Weingärtner, Tobias Pinsdorf, Kyrill S. Rogacev, Lutz Blömer, Yvonne Grenner, Stefan Gräber, Christof Ulrich, Matthias Girndt, Michael Böhm, Danilo Fliser, Ulrich Laufs, Dieter Lütjohann, Gunnar H. Heine

**Affiliations:** 1 Klinik für Innere Medizin III, Kardiologie, Angiologie und Internistische Intensivmedizin, Universitätsklinikum des Saarlandes, Homburg/Saar, Germany; 2 Institut für Klinische Chemie und Klinische Pharmakologie, Universitätsklinikum Bonn, Bonn, Germany; 3 Institut für Medizinische Biometrie, Epidemiologie und Informatik, Universitätsklinikum des Saarlandes, Homburg/Saar, Germany; 4 Klinik für Innere Medizin II, Universitätsklinikum des Saarlandes, Homburg/Saar, Germany; 5 Klinik für Innere Medizin IV, Nephrologie und Hochdruckkrankheiten, Universitätsklinikum des Saarlandes, Homburg/Saar, Germany; University of Tor Vergata, Italy

## Abstract

**Background:**

The relationship of cholesterol homeostasis and carotid intima-media thickness (cIMT) is unknown. To address this, we assessed markers of cholesterol homeostasis (serum plant sterols and cholesterol precursor concentrations as surrogate measures of cholesterol absorption and synthesis, respectively) and cIMT in a middle-aged, statin-naive population.

**Methods:**

In this prospective study of primary prevention cIMT was measured by ultrasound in 583 hospital employees aged 25–60 years without prevalent cardiovascular disease or lipid-modifying medication. The serum concentrations of plant sterols (as markers of cholesterol absorption) were measured by gas-liquid chromatography. Lathosterol serum concentrations were quantitated to assess hepatic cholesterol synthesis.

**Results:**

cIMT correlated positively with serum cholesterol (r = 0.22, *P*<0.0005) and lathosterol-to-cholesterol (r = 0.18, *P<0.001*). In contrast, plant sterols, as markers of cholesterol absorption, showed a weak negative correlation to cIMT measurements (r = −0.18; *P<0.001* for campesterol-to-cholesterol). Stratifying subjects by serum sterol levels, we found that cIMT increased continuously over quintiles of serum cholesterol (*P*<0.0005) and was positively associated to serum lathosterol-to-cholesterol levels (*P = *0.007), on the other hand, plant sterol levels showed a weak negative association to cIMT (*P<0.001* for campesterol-to-cholesterol).

**Conclusions:**

In this population without prevalent cardiovascular diseases or lipid-modifying medication, markers of increased endogenous cholesterol synthesis correlated positively with cIMT, while markers of cholesterol absorption showed a weakly negative correlation. These data suggest that not only total serum cholesterol levels but also differences in cholesterol homeostasis are associated with cIMT.

## Introduction

Plasma cholesterol is derived from enteral cholesterol absorption and hepatic cholesterol synthesis,[1]–[3] and represents a causal factor for the development of atherosclerotic diseases [4], [5]. Serum concentrations of plant sterols that are exclusively of dietary origin, such as sitosterol and campesterol, reflect the efficacy of cholesterol absorption [3], [6]. Lathosterol, an intermediate of cholesterol synthesis, is a marker of hepatic cholesterol formation [1], [2]. Clinical trials of primary and secondary prevention have clearly demonstrated that inhibiting cholesterol synthesis with statin treatment reduces the risk of cardiovascular events and mortality [7], [8]. In contrast, the effect of inhibition of cholesterol absorption by plant sterol-supplemented functional foods (e.g. margarine supplemented with plant sterol esters), by the NPC1L1-inhibitor ezetimibe, or by the administration of bile acid resins for the primary prevention of atherosclerotic lesion development is unknown [9]–[13].

Ultrasound of the carotid arteries allows clinicians to detect the development of atherosclerosis at an early stage. Measurements of carotid intima-media-thickness (cIMT), which is a marker of future cardiovascular events [14]–[17], have been applied to assess the effectiveness of lipid-lowering treatments in vascular disease [18], [19]. However, the differential effects of cholesterol absorption and synthesis on cIMT are unknown [20].

In the prospective I LIKE HOMe study (Inflammation, Lipoprotein Metabolism, and Kidney Damage in early atherogenesis—The Homburg Evaluation), we evaluated cholesterol absorption and synthesis in relation to cIMT in 583 middle-aged hospital employees. Participants had no known cardiovascular disease (CVD), and were not taking lipid-modifying medication [21].

## Methods

### Subjects

Healthcare workers at the Saarland University Hospital in Homburg/Saar, Germany, aged 25–60 years who were scheduled to undergo routine medical checkups were invited to have an ultrasonographic examination of their carotid arteries. Participants were excluded if they had prevalent CVD, diabetes mellitus, active tumor disease, inflammatory/autoimmune disease requiring systemic immunosuppressive treatment, stage 4 or stage 5 chronic kidney disease, were strict vegetarians or were on any lipid-modifying medications. Prevalent CVD was diagnosed in subjects who had a history of myocardial infarction, coronary artery angioplasty, stenting, bypass surgery, major stroke, carotid endarterectomy, non-traumatic lower extremity amputation, lower limb artery bypass surgery, or angioplasty. Subjects on antihypertensive medication, hormone replacement therapy, or oral contraceptives were allowed to enter the I LIKE HOMe study.

A total of 583 participants met these inclusion criteria and were included in the analysis. Written informed consent was obtained from all study participants. The study protocol was approved by the Ethics Committee of the University of Saarland. A standardized questionnaire was used to record patient histories of smoking, diabetes, current drug intake, cardiovascular comorbidity, and premature onset of CVD in the family (defined as myocardial infarction or stroke before the age of 60 years in first-degree relatives). Anthropomorphometric measurements and resting blood pressure were recorded. Body mass index (BMI) was calculated as the individual's body weight divided by their height squared. Systolic blood pressure (SBP), diastolic blood pressure (DBP), and heart rate were measured after 5 min of rest. Mean blood pressure was calculated as DBP+ [(SBP-DBP)/3].

Participants were categorized as active smokers if they were current smokers or had stopped smoking less than 1 month before entry into the study. Individuals who had self-reported diabetes mellitus, a non-fasting blood sugar level of >200 mg/dl, a fasting blood sugar level of >126 mg/dl, or were currently using hypoglycemic medication were categorized as diabetic and excluded from the study.

### Biochemical Analysis

Blood samples were taken from all subjects under standardized conditions. Plasma glucose, creatinine, total cholesterol, and high-density lipoprotein (HDL) cholesterol were obtained using standard techniques. Gas chromatography-flame ionization analysis was performed for quantification of cholesterol and mass spectrometry was performed for lathosterol and plant sterols as described previously [22].

### Carotid Ultrasound

The cIMT of the common carotid artery was measured from high-resolution, two-dimensional ultrasound images obtained using a linear-array 7.5 MHz transducer (Sonoline Siena, Siemens, Erlangen, Germany). With the subject in a supine position and the head slightly extended and turned in the opposite direction, longitudinal B-mode images of the distal common carotid artery and the carotid bulb were acquired and digitally stored for offline reading by a single blinded investigator. The cIMT was defined as the distance between the leading edge of the lumen interface and the media-adventitia interface of the far wall. Three representative measurements were calculated for the far wall of both common carotid arteries at predefined positions (1.0, 2.0, and 3.0 cm proximal to the bifurcation), and these six IMT readings were averaged to give the mean common cIMT. The cIMT was not measured at the site of a carotid plaque, which was defined as a focal thickening of the intima-media complex ≥1.1 mm irrespective of echogenicity.

### Statistical Analysis

Data management and statistical analyses were performed using SPSS 13.0. Unless otherwise indicated, continuous data are expressed as the mean ± standard deviation (SD) and compared by the Mann-Whitney *U* test. Categorical variables are presented as the percentage of participants. Correlation coefficients were calculated by the Spearman test. A value of *P<*0.05 was considered statistically significant. Subjects were stratified into quintiles of serum cholesterol and non-cholesterol sterol levels. We recorded data on cIMT measurements, age, BMI, and lipid metabolism parameter trends across increasing quintiles of cholesterol, lathosterol, campesterol, and sitosterol using one-way analysis of variance (ANOVA) and partitioning the between-group sums of squares into trend components. Multivariable regression analyses were performed to further analyze markers of cholesterol metabolism in relation to cIMT. The Sobel test was used to assess whether the association between BMI and cIMT was partially mediated by lathosterol.

## Results

### Baseline Characteristics of Subjects Enrolled in the Study

The baseline characteristics of the 583 subjects enrolled in the study are shown in Table 1. The participants included 376 females (64.5%), 105 individuals with a family history of premature-onset CVD (18.0%), and 181 current smokers (31.1%). According to the Framingham risk score 94.5% of the total study population was classified as “low cardiovascular risk”.

**Table 1:** Patient Characteristics.

Patient Characteristics	Mean ± SD
N	583
Age (years)	42±8
Male/Female	207/376
Current or ex-smoker	181
Family history	105
Body mass index (kg/m^2^)	25.0±4.4
Framingham risk score <10%	94.50%
Framingham risk score 10–20%	5%
Framingham risk score >20	0.50%
Heart rate (per min)	73.7±10.3
Mean blood pressure (mmHg)	100.0±12.0
Serum glucose (mg/dl)	91.4±15.3
Glomerular filtration rate (ml/min/1.73 m^2^)	86.7±14.9
IMT (mm)	0.430±0.077
Total cholesterol (mg/dl)	204.7±37.2
LDL cholesterol (mg/dl)	112.9±34.4
HDL cholesterol (mg/dl)	67.1±17.0
Triglycerides (mg/dl)	119.9±82.4
Lathosterol (mg/dl)	0.29±0.13
Campesterol (mg/dl)	0.39±0.19
Sitosterol (mg/dl)	0.29±0.12
Lathosterol-to-cholesterol ratio (µg/mg)	1.39±0.56
Campesterol-to-cholesterol ratio (µg/mg)	1.91±0.85
Sitosterol-to-cholesterol ratio (µg/mg)	1.42±0.52

### The Relationship of cIMT to Subject Baseline Characteristics

**Figure 1:**
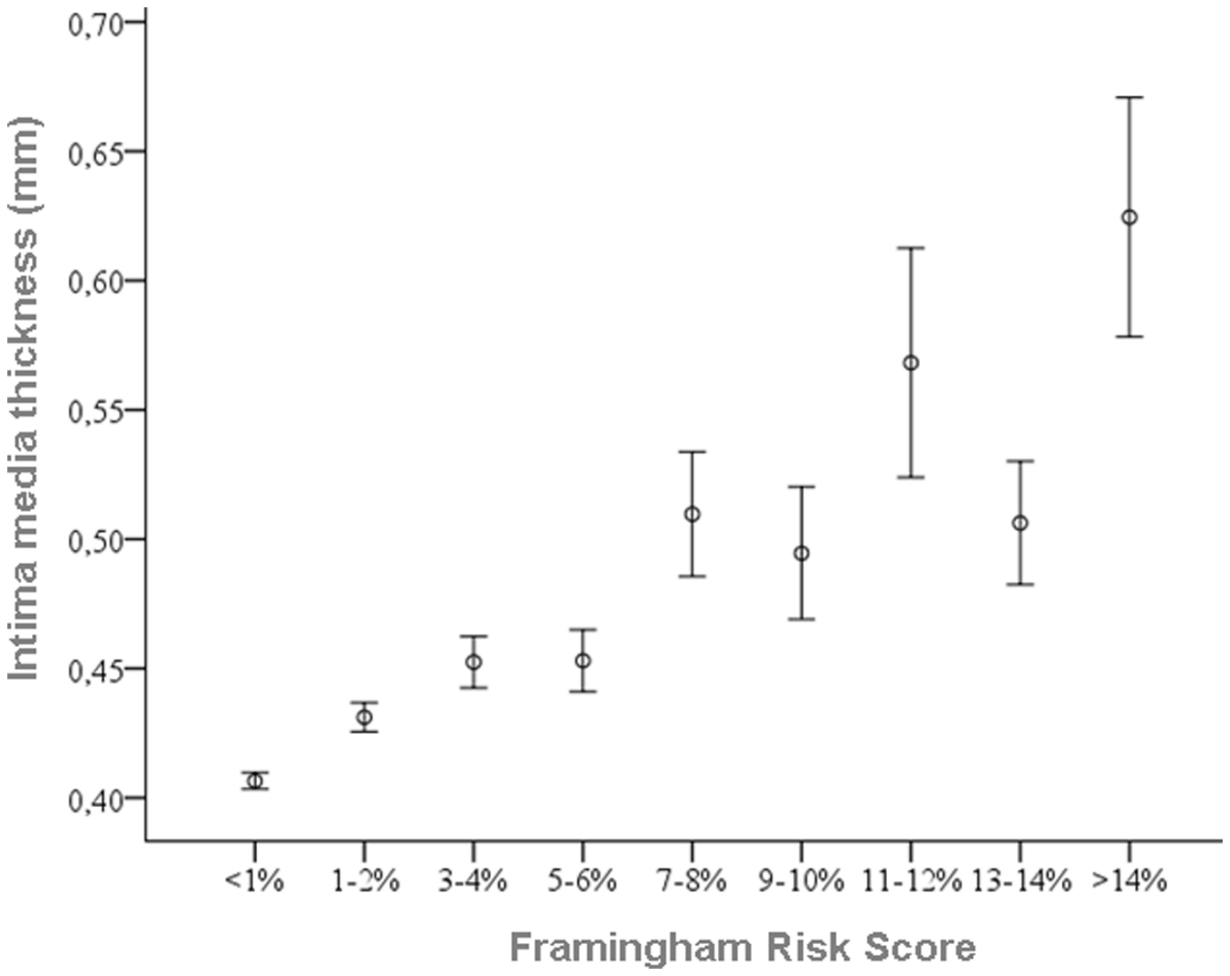
Intima-media thickness by categories of risk for coronary heart disease. Values are mean +/− SEM.

For the entire study cohort, cIMT was positively correlated with BMI (r = 0.35, *P*<0.0005), age (r = 0.52, *P*<0.0005), mean blood pressure (r = 0.40, *P*<0.0005), plasma low density lipoprotein (LDL) cholesterol (r = 0.24, *P*<0.0005) and triglycerides (r = 0.21, *P*<0.0005). The plasma level of HDL cholesterol was negatively correlated to cIMT (r = −0.19, *P*<0.0005). As a consequence, we found a significant association between cIMT and Framingham risk score-based categories of risk for coronary heart disease (CHD) (Fig. 1).

### The Relationship of Non-cholesterol Sterols to Clinical Parameters

BMI was associated with non-cholesterol sterols and their ratios to cholesterol. While sitosterol (r = −0.25, *P*<0.0005), the sitosterol-to-cholesterol ratio (r = −0.34, *P*<0.0005), campesterol (r = −0.27, *P*<0.0005), and the campesterol-to-cholesterol ratio (r = −0.34, *P*<0.0005) were negatively correlated with BMI, markers of endogenous cholesterol synthesis, lathosterol, and the lathosterol-to-cholesterol ratio, showed a positive correlation with BMI (r = 0.43, *P*<0.0005 and r = 0.42, *P*<0.0005, respectively). Furthermore, blood pressure was positively correlated with lathosterol (r = 0.36, *P*<0.0005) and lathosterol-to-cholesterol ratio (r = 0.26, *P*<0.0005), but correlated only weakly, if at all, with cholesterol absorption (sitosterol r = −0.06, *P* = n.s.; sitosterol-to-cholesterol r = −0.22, *P*<0.0005; campesterol r = −0.09, *P*<0.05; campesterol-to-cholesterol r = −0.22, *P*<0.0005). Age was weakly correlated with lathosterol (r = 0.22, *P*<0.005) and lathosterol-to-cholesterol ratio (r = 0.10, *P*<0.012), but not with sitosterol or campesterol. As a result, the Framingham risk score correlated with lathosterol (r = 0.28, *P<0.0005*) and the lathosterol-to-cholesterol ratio (r = 0.17, *P<0.0005*), but correlated only weakly, if at all, with campesterol (r = 0.03, *P = n.s.*) and the campesterol-to-cholesterol ratio (r = −0.10; P<0.021) and sitosterol (r = 0.04, *P = n.s.*) and the sitosterol-to-cholesterol ratio (r = −0.20, P<0.004).

### The Relationship of cIMT to Serum Cholesterol

**Figure 2:**
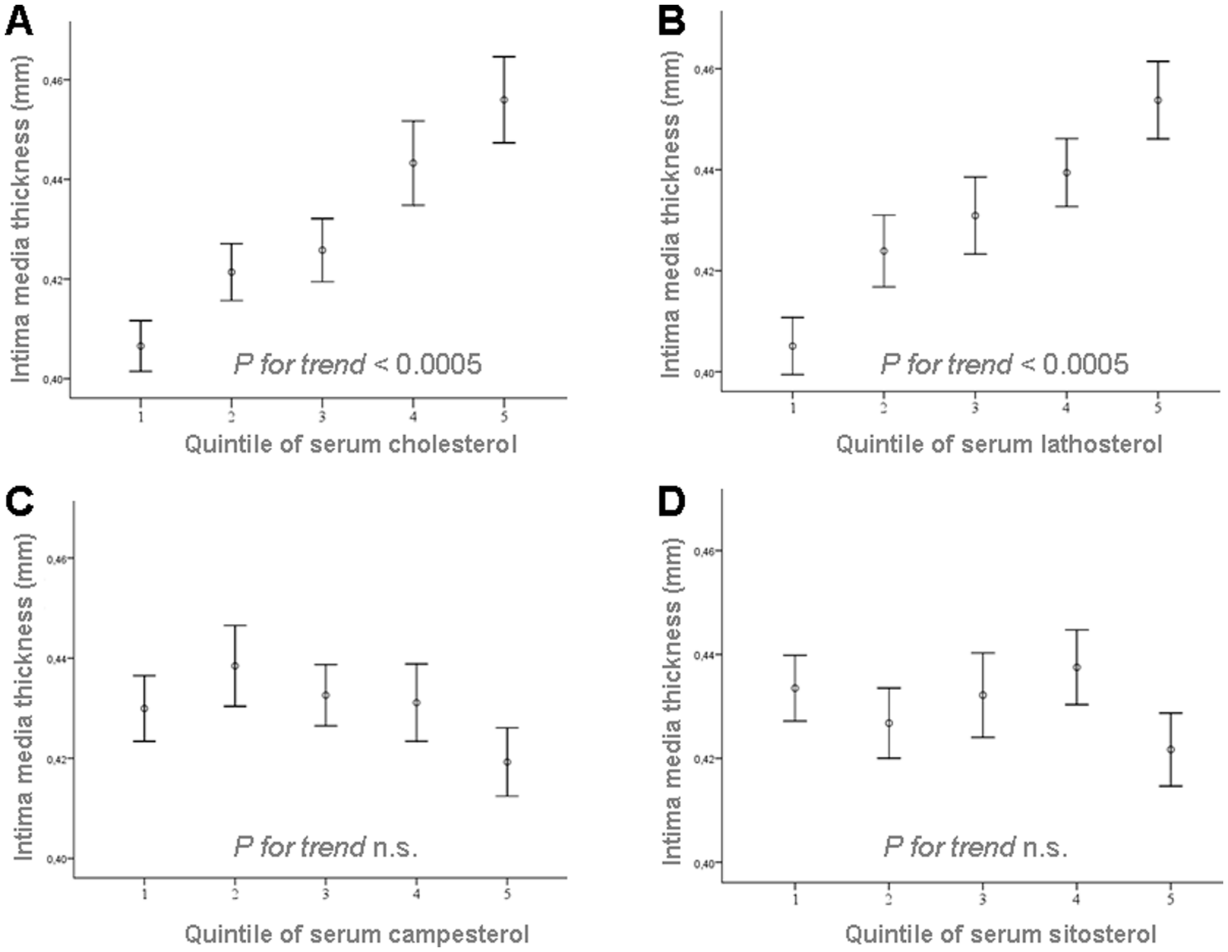
Intima-media thickness measurements in relation to quintiles of serum cholesterol, lathosterol, campesterol and sitosterol. A: Intima-media thickness measurements in relation to quintiles of serum cholesterol concentration (*P<0.0005*). Values are mean +/− SEM. B: Intima-media thickness measurements in relation to quintiles of serum lathosterol concentration (*P<0.0005*). Values are mean +/− SEM. C: Intima-media thickness measurements in relation to quintiles of serum campesterol concentration. Values are mean +/− SEM. D: Intima-media thickness measurements in relation to quintiles of serum sitosterol concentration. Values are mean +/− SEM.

There was a positive correlation between cIMT and serum cholesterol level (r = 0.22 *P*<0.001). Stratifying subjects into quintiles based on serum cholesterol level, we found that cIMT measurements increased continuously over quintiles of serum cholesterol (*P for trend*; *P*<0.0005) with subjects in quintiles 5 and 4 having higher cIMT measurements than those in quintile 1 (0.46±0.09 mm and 0.44±0.09 mm vs. 0.41±0.06 mm, *P*<0.005 and *P*<0.0005, respectively; Fig. 2A). Further analysis of the distribution of subject characteristics with serum cholesterol revealed that age increased significantly across quintiles. However, in a partial correlation analysis controlling for age, the association between cIMT and cholesterol remained statistically significant. Moreover, BMI, lipid subfractions (LDL cholesterol, HDL cholesterol, and triglycerides), and all non-cholesterol sterols (campesterol, sitosterol, and lathosterol) increased over the quintiles of serum cholesterol levels, while the non-cholesterol sterol-to-cholesterol ratios did not (data not shown).

### The Relationship of cIMT to Endogenous Cholesterol Synthesis

**Figure 3:**
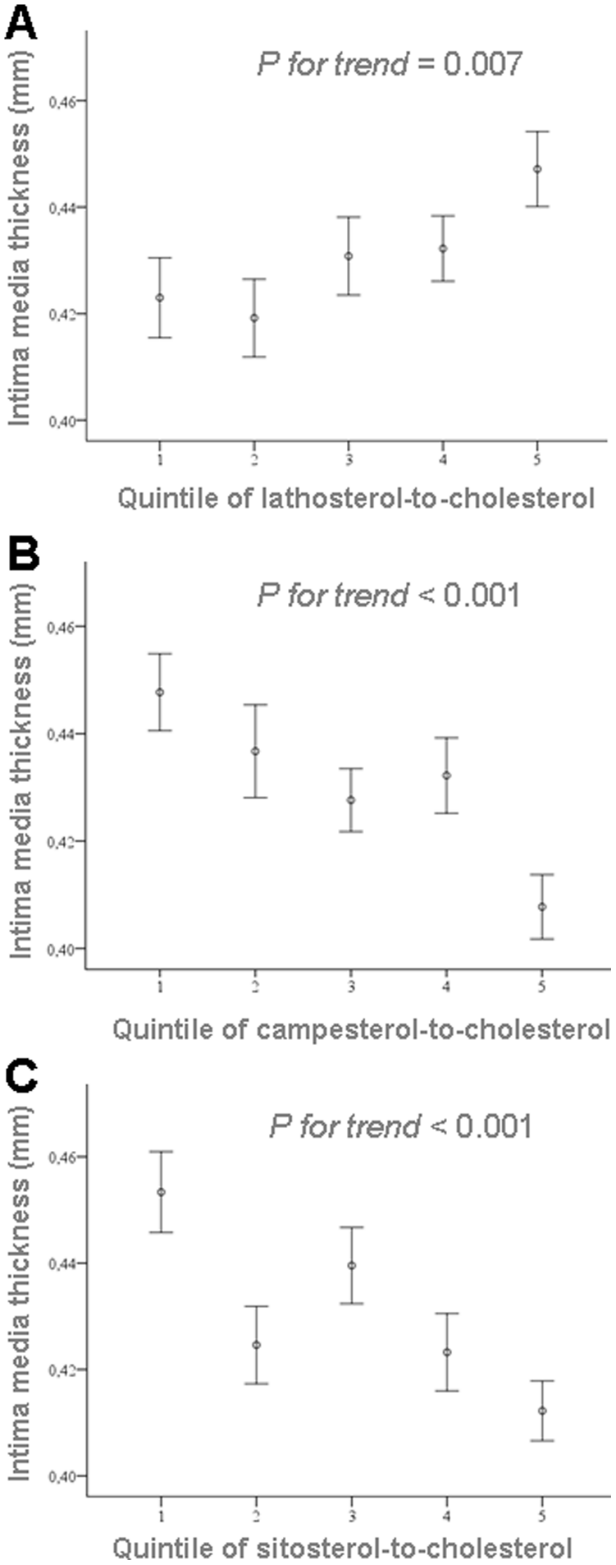
Intima-media thickness measurements in relation to quintiles of serum lathosterol-to-cholesterol, campesterol-to-cholesterol and sitosterol-to-cholesterol. A: Intima-media thickness measurements in relation to quintiles of lathosterol-to-cholesterol (*P = 0.007*). Values are mean +/− SEM. B: Intima-media thickness measurements in relation to quintiles of campesterol-to-cholesterol (*P<0.001*). Values are mean +/− SEM. C: Intima-media thickness measurements in relation to quintiles of sitosterol-to-cholesterol (*P<0.001*). Values are mean +/− SEM.

cIMT measurements correlated positively with serum lathosterol (r = 0.25, *P*<0.001) and lathosterol-to-cholesterol ratio (r = 0.18, *P*<0.001) as a surrogate for endogenous cholesterol synthesis. The latter association was found in both men (r = 0.164; p = 0.018) and women (r = 0.153; p = 0.003). After stratification into quintiles by serum lathosterol levels, cIMT measurements increased continuously over quintiles of serum lathosterol (*P for trend; P*<0.0005) with significantly higher values in quintiles 5 and 4 than in quintile 1 (0.45±0.08 mm and 0.44±0.07 mm vs. 0.41±0.06 mm, *P*<0.005 and *P*<0.0005, respectively; Fig. 2B). A similar relation could be observed after stratification into quintiles by lathosterol-to-cholesterol levels *(P for trend; P = 0.007*; Fig. 3A). Similarly, age increased across the quintiles of serum lathosterol. Again, the association between cIMT and lathosterol remained significant in a partial correlation analysis controlling for age. Total cholesterol, LDL cholesterol, triglycerides, and BMI increased over the lathosterol quintiles, while non-cholesterol sterols (campesterol and sitosterol), their ratios to cholesterol, and HDL cholesterol decreased (data not shown).

### The Relationship of cIMT to Cholesterol Absorption Markers

In contrast to serum cholesterol and the cholesterol precursor lathosterol, serum levels of the plant sterol campesterol and the campesterol-to-cholesterol ratio showed a weakly negative correlation with cIMT (r = −0.08, *P*<0.04, and r = −0.18, *P*<0.001, respectively). The absolute concentration of sitosterol did not correlate with cIMT (r = −0.05, *P*<0.27); the sitosterol-to-cholesterol ratio showed a weakly negative correlation with cIMT (r = −0.17, *P*<0.0005). Again, the association of cIMT measurements with the campesterol-to-cholesterol ratio and sitosterol-to-cholesterol ratio were found in both men and women (data not shown).

Carotid intima-media thickness did not differ significantly with absolute campesterol or sitosterol levels after stratification into quintiles by plant sterol levels (Fig. 2C and Fig. 2D), but showed a weak negative correlation across quintiles of campesterol-to-cholesterol (*P for trend; P<0.001*; Fig. 3B) and sitosterol-to-cholesterol (*P for trend; P<0.001*; Fig. 3C). Across quintiles of both cholesterol absorption markers, plasma levels of total cholesterol, LDL cholesterol, and HDL cholesterol increased, while BMI, serum lathosterol concentration and lathosterol-to-cholesterol ratio decreased (data not shown).

### Multivariable Regression Analysis of Markers of Cholesterol Metabolism in Relation to cIMT

Multivariable regression analyses revealed that elevated serum lathosterol and low serum campesterol and sitosterol are associated with cIMT after adjusting for age and LDL-cholesterol (Table 2). When BMI was also adjusted for, no marker of cholesterol metabolism remained independently associated with cIMT. Nonetheless, the Sobel test revealed that the impact of BMI on cIMT is mediated by lathosterol. BMI remains independently associated with cIMT after further adjustment for mean arterial pressure. A final multivariable model which included all parameters assessed failed to show an independent association between cIMT and either BMI or lathosterol probably due to multicollinearity of the diverse markers of lipid metabolism.

**Table 2:** Multivariable regression analysis of cholesterol and markers of cholesterol metabolism in relation to cIMT.

	Univariable		adjusted for LDL-c and age		adjusted for LDL-c, age and BMI		adjusted for LDL-c, age, BMI and mean BP		adjusted for LDL-c, age, BMI, mean BP, HDL-c and TG	
	B	*P-value*	B	*P-value*	B	*P-value*	B	*P-value*	B	*P-value*
Lathosterol	0.221	<0.001	0.088	0.026	0.023	0.582	0.003	0.950	−0.004	0.910
Campesterol	−0.055	0.187	−0.068	0.077	−0.006	0.885	−0.004	0.916	0.029	0.483
Sitosterol	−0.035	0.397	−0.080	0.038	−0.018	0.648	−0.014	0.725	0.020	0.621
BMI	0.325	<0.001	0.186	<0.001	(not applicable)		0.096	0.018	0.050	0.233

B: standardized regression coefficient; *P*: level of significance. All values have been log-transformed prior to analysis.

LDL-c: low density lipoprotein cholesterol; HDL-c: high density lipoprotein cholesterol; BP: blood pressure; BMI: body mass index; TG: triglycerides.

## Discussion

The present data demonstrate that both total serum cholesterol levels and markers for endogenous cholesterol synthesis correlate positively with cIMT in a middle-aged statin-naïve population. In contrast, plant sterols used as markers of cholesterol absorption showed a weakly negative correlation with cIMT. The reciprocal relation of cholesterol synthesis and cholesterol absorption markers further strengthens the data and suggests that not only total serum cholesterol levels but also differences in cholesterol homeostasis are associated with the development of atherosclerosis.

Carotid IMT is the most commonly used and best validated ultrasound measure of early and intermediate stages of atherosclerosis, including in young populations without known CVD.[23] In fact, evidence suggests that this association may be particularly strong in younger individuals with low cardiovascular risk [17]. Furthermore, cIMT measurements have been useful as a surrogate parameter to assess treatment efficacy in lipid-lowering trials [10], [19].

In addition to serving as a marker molecule for enteral cholesterol absorption, the understanding of autosomal recessive sitosterolemia led to speculation that plant sterols per se represent a cardiovascular risk factor [24], [25]. Elevated plant sterol concentrations, xanthomatosis, and premature, frequently lethal atherosclerosis in young subjects are the most striking features in patients with homozygous sitosterolemia. These features are due to mutations of the gene locus for the ABCG5 and ABCG8 co-transporters, which result in increased absorption and reduced biliary elimination of all sterols [26], [27]. The fact that patients with this disease present with an aggressive vascular disease process despite nearly normal cholesterol levels raised the question of whether high phytosterol levels exert atherogenic effects [28]. This hypothesis has been supported by the finding that plant sterols are detected in atherosclerotic lesions from individuals with apparently normal cholesterol absorption [29]. Furthermore, some epidemiological studies suggest that increased cholesterol absorption with upper normal plasma plant sterol concentrations might reflect increased vascular risk in non-sitosterolemic subjects [30]–[37]. On the other hand, other well performed studies do not show such an association; neither a recent case-control study nested into the EPIC Norfolk population study, nor data from the LASA study demonstrated a positive relationship between plasma plant sterol levels and cardiac risk [38], [39].

The findings of our study imply that there is no positive association between serum plant sterol concentrations and cIMT measurements in a middle-aged population with low plant sterol serum levels. However, this relation may be different in populations with higher cardiovascular risk and in later stages of CVD. Notably, in the first epidemiological study to report an association of plant sterol levels and increased cardiovascular risk, Glueck and colleagues have already found that this relation was especially strong in individuals with a positive family history, high serum cholesterol levels, and generally higher cardiovascular risk [30]. Similar findings have been reported by Sudhop and colleagues in patients scheduled for coronary artery bypass surgery [32], and by Strandberg and colleagues in a population of home-dwelling elderly individuals [34]. Another recently published epidemiological study indicated that increased campesterol-to-cholesterol and sitosterol-to–cholesterol ratios were associated with coronary artery disease severity [35]. In the Framingham Off-Spring Study, higher absorption marker concentrations and lower synthesis markers were significant predictors of CVD [36], a finding that is in line with data reported by our research group in patients with known severe aortic stenosis [37]. Interestingly, similar to Matthan and colleagues, we found that, in patients with severe aortic stenosis and an average age of 70 years, cholesterol homeostasis shifted towards high cholesterol absorption and low cholesterol synthesis was a better predictor of concomitant coronary artery disease than any other established cardiovascular risk factor. Furthermore, data from the PROCAM study demonstrate that upper normal plant sterol serum levels do not affect cardiovascular risk in individuals with low and intermediate cardiovascular risk, but increase cardiovascular risk three-fold in individuals in the highest tertile [33]. Therefore, Assmann and colleagues concluded that plant sterols as a marker for increased cholesterol absorption are associated with an increased cardiovascular risk, especially in individuals who have an increased cardiovascular risk according to the PROCAM score. In this regard, the findings of this study are in line with PROCAM data showing that serum plant sterol concentrations do not correlate with preclinical atherosclerosis in individuals who have low cardiovascular risk in primary prevention setting. Finally, Strandberg and colleagues followed mildly hypercholesterolemic but otherwise healthy men for 22 years [40] and found that increased serum levels of plant sterols were associated with strongly decreased cardiovascular risk. Notably, the study population assessed by Strandberg and colleagues was in their forties, similar in age to the I LIKE HOMe participants, while cohort studies reporting that high plant sterol levels predict adverse outcomes recruited mostly older subjects. Strandberg and coworkers therefore hypothesized that these controvert findings might be due to selection, as susceptible individuals with high cholesterol synthesis and low cholesterol absorption (which reflect metabolic syndrome), as well as increased BMI, may die before reaching old age. Therefore, Strandberg concluded that the relationship between non-cholesterol sterols and cardiovascular risk might be another example of “reverse epidemiology” [41]. This concept states that factors harmful in earlier life – such as overweight (the “obesity paradox”), blood pressure, and cholesterol – may paradoxically appear beneficial in older age. The present study population would therefore represent a phase in which higher cholesterol absorption and lower cholesterol synthesis each appear to be protective.

### Limitations

The dietary habits of study participants were not controlled in detail prior to study enrollment. We therefore cannot exclude that variations in dietary habits influenced the amount of ingested plant sterols. However, previous work has supported the notion that even a vegetarian lifestyle does not have major effects on plasma plant sterol concentrations [42]. Moreover, interpretation of the data is complicated by the fact that cholesterol metabolism is closely related to features of the metabolic syndrome and obesity [43]. Additionally, lipoproteins of hepatic and intestinal origin (specifically, apolipoprotein B100 and apolipoprotein B48) have not been assessed in this study. Finally, a long-term follow-up is needed to determine whether differences in cholesterol absorption and synthesis markers correlate with clinical events.

### Conclusions

In this middle-aged population without prevalent CVD or lipid modifying medication, markers for endogenous cholesterol synthesis, but not markers for cholesterol absorption, were positively related to cIMT as a measurement for subclinical atherosclerosis. Because clinical trials evaluating novel cardiovascular therapies to reduce serum cholesterol levels use cIMT measurements as surrogate endpoints, these findings are of importance and may have to be considered in the design of future clinical trials investigating this issue. Furthermore, these data suggest that in a middle-aged population cholesterol homeostasis shifted towards increased markers for cholesterol synthesis, rather than cholesterol absorption (a constellation typical for metabolic syndrome) is associated with increased cardiovascular risk.
